# ParallelStructure: A R Package to Distribute Parallel Runs of the Population Genetics Program STRUCTURE on Multi-Core Computers

**DOI:** 10.1371/journal.pone.0070651

**Published:** 2013-07-29

**Authors:** Francois Besnier, Kevin A. Glover

**Affiliations:** 1 Department of Population Genetics, Institute of Marine Research, Bergen, Norway; 2 Department of Informatics, Faculty of Mathematics and Natural Sciences, University of Bergen, Bergen, Norway; Swiss Federal Institute of Technology (ETH Zurich), Switzerland

## Abstract

This software package provides an R-based framework to make use of multi-core computers when running analyses in the population genetics program STRUCTURE. It is especially addressed to those users of STRUCTURE dealing with numerous and repeated data analyses, and who could take advantage of an efficient script to automatically distribute STRUCTURE jobs among multiple processors. It also consists of additional functions to divide analyses among combinations of populations within a single data set without the need to manually produce multiple projects, as it is currently the case in STRUCTURE. The package consists of two main functions: *MPI_structure*() and *parallel_structure*() as well as an example data file. We compared the performance in computing time for this example data on two computer architectures and showed that the use of the present functions can result in several-fold improvements in terms of computation time. *ParallelStructure* is freely available at https://r-forge.r-project.org/projects/parallstructure/.

## Introduction

The software package STRUCTURE was introduced in 2000 by Pritchard et al. [1], and has become one of the most widely used population genetic programs of the last decade. STRUCTURE has brought outstanding contributions to the fields of population genetics and molecular ecology by providing a user friendly tool for analyzing multi-locus genotype data to address evolutionary questions such as population structure, hybridization, or population admixture e.g., [2]-[6].

STRUCTURE analysis relies on multiple MCMC re-sampling and it is therefore time consuming when it involves data sets with large numbers of individuals, populations, and loci. Adding to the time of analysis is the fact that the program is often run with many iterations for each number of genetic clusters to provide robust data for implementation of post-hoc methods to determine the number of populations in the data set [7]. In general, one efficient way to speed up computing processes is to distribute tasks on several computing units (core/CPU). This solution is becoming commonplace since shared memory multi-core processors are widely available on the market. Even common laptops are usually equipped with, at least, dual or quad-core processors, and up to 8 cores are becoming the standard.

Even though STRUCTURE does not support native multi processor tasking, it is possible to manually run STRUCTURE analyses in parallel on multiple CPUs by simply opening several graphic interface windows at the same time. Indeed, the analysis of genetic data with STRUCTURE usually involves multiple independent runs, it is thus straightforward to simply distribute the *N* runs to be performed on *n* available parallel computing cores. However, manual parallel computing in STRUCTURE is a suboptimal solution, as it requires frequent monitoring from the user who needs to distribute the tasks manually among the available processors. Furthermore, when the user wants to analyze different combinations of populations within a data set, separate analyses have to be manually established on separate data files for each combination of populations. A much more effective way to distribute jobs among parallel processors is to use scripts to run STRUCTURE on the command line version.

Using script programming can make more efficient use of multi-core processors by automatically distributing analyses to all the available cores/CPUs and renewing the task of each core/CPU as soon as a given job is completed. This solution is more efficient but requires specific script and parallel programming skills.

The present package *ParallelStructure* provides a R [8] framework to run genetic analysis in STRUCTURE and make an efficient use of multi-core processors. R is an extremely popular support for population genetics tools e.g., [9]-[11], and an increasing number of programs have started to incorporate R functions to produce graphic outputs, such as the latest versions of BAYESCAN [12] or ARLEQUIN [13]. It is thus likely that many STRUCTURE users are already familiar with the R environment. Here, we introduce the package *ParallelStructure* that is intended for users aiming to run intensive analyses of their data sets (e.g. by making large number of replicates and/or testing many values of K), and willing to use STRUCTURE from a R function instead of the graphic interface in order to efficiently distribute a set of pre-defined jobs on a multi-core / multiprocessor computer. Furthermore, when dealing with large data sets, *ParallelStructure* can make use of one single data file from which the intended populations can be selected, thus avoiding the need of creating multiple smaller data files for each analysis in particular.

*ParallelStructure* consists of a R script that imports STRUCTURE command line options into a R function, and runs several STRUCTURE analyses in parallel by using either Rmpi package [14] or parallel package [8].

## Materials and Methods

### Package description

*ParallelStructure* package consists of two main functions, *MPI_structure*() and *parallel_structure*() as well as an example data file, corresponding *joblist* file and a user manual (file S1). The two functions *MPI_structure*() and *parallel_structure*() are equivalent as they perform the same task and work with the same input file and parameter set. The difference between them is the method that the function relies on for distributing jobs among CPUs: *MPI_structure*() relies on the R package *Rmpi* whereas *parallel_structure*() relies on *mclapply* function from *parallel* package. Rmpi is distributed in the CRAN repository for MacOS, but requires manual installation under Windows. *Parallel* package is distributed with R since version 2.14.0 but is still under development. *parallel_structure*() might not be fully functional under Windows architecture, and should not be used in GUI or embedded environments as it may cause crashes. We thus strongly recommend to use *MPI_structure*() by default.

A list of tasks to be performed is stored in a *joblist* file in which each line corresponds to an individual job. While STRUCTURE input format requires a different data file for each set of populations, *ParallelStructure* offers the possibility to work from a large input file containing all the populations one might need to analyze. This avoids making a different input file for each set of populations to be analyzed. Instead, the user defines, in each job, the set of populations to be included as well as parameter *K*, burnin and number of iterations. If all the populations in the data set are to be analyzed pairwise (*all vs. all*), the list of populations for the given job can be replaced by ”pairwise. matrix”, e.g. job T11 in example *joblist*.

### Example data set

The example file provided with the package contains microsatelite data on nine loci for 987 individuals divided in 8 populations. The *joblist* given with the example consists of 20 jobs for which a variable set of populations from the eight present in the dataset are included. With the exception of job T11, the list of populations to be included is given as a comma-separated list of the population’s id as they appear in the input data file. For job T11, the list of populations was replaced by the character string “pairwise. matrix”. In this case, all populations are analyzed against each other producing a total of *n*(*n*-1)/2 jobs, where *n* is the total number of populations in the dataset. Output files can be stored in a dedicated directory specified by the user. After executing a list of jobs, ParallelStructure writes a. csv formatted summary file in the working directory. This file contains for each job, the job ID, main parameters, and the following summary statistics: log-likelihood of the data, mean and variance of the likelihood as well as mean value of alpha. The function argument “printqhat=1” and “plot_output=1” also give the possibility to generate graphic output as. pdf files. In such a case, one graphic file is generated for each job.

### Time of execution

The execution time was compared for the example data set, i.e., microsatelite genotypes for nine loci for 987 individuals divided in 8 populations. The set of jobs consisted of 19 STRUCTURE runs with various number of population sub-sets, with 1000 burnin and 10000 iterations. One full pair-wise matrix job (Job T11) that runs STRUCTURE for each pair of populations in the dataset was also included in the list of jobs. Execution time was computed on: (a) a Windows 7 laptop PC equipped with a Core i7 2.2GHz quad core processor with 8Gb of RAM, (b) an Apple workstation equipped with an Intel Xeon 2.26GHz double quad core processor with 16GB of RAM. Both computer architecture were running on their respective operating system: Windows 7 and MAC OS X respectively, as well as on one common operating system for both architecture: Linux Ubuntu 12.04. To assess the gain of computation time obtained by parallelization, we computed the speed up as S_p_=T_1_/T_p_, where S_p_ is the speed-up obtained by distributing a task on p processors, *T*_*1*_ is the execution time on one processor (sequential algorithm), and *T*_*p*_ the execution time of the task on *p* processors.

## Results and Discussion

For the performance comparison, as much as twice the number of physical processor cores in each respective architecture were used (i.e., 8 cores for the Intel i7 architecture, and 16 cores for the Intel Xeon architecture). This was to make use of hyperthreading technology (duplicating physical processor cores into two virtual cores) that is available on both architectures.

Execution time of the sequential tasks (on only one processor) on the Intel i7 architecture required respectively 640 sec and 462 sec running on Windows 7 and Linux Ubuntu. On the Intel Xeon architecture, execution time was 630 sec and 613 sec running on Mac OS X or Linux Ubuntu respectively. When comparing the two architectures on the same operating system, the newer i7 processor was approximately 25% faster than the older Intel Xeon.

**Figure 1:**
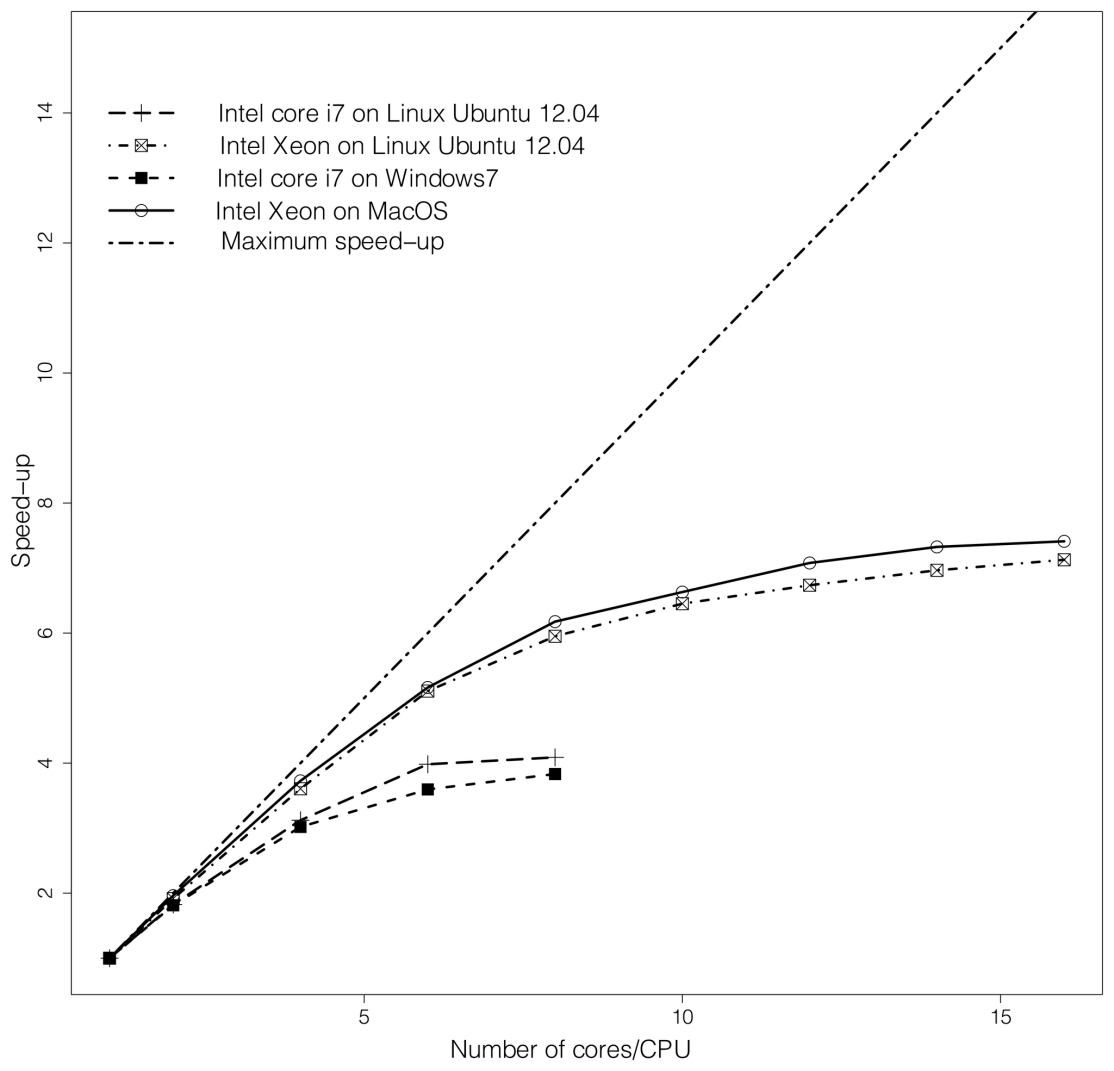
Gains in job execution time (speed-up) for the example data when running on variable number of processors on two computer architectures. (a) a Windows 7 laptop PC equipped with a Core i7 2.2GHz quad core processor with 8Gb of RAM and (b) an Apple workstation equipped with an Intel Xeon 2.26GHz double quad core processor with 16GB of RAM. Maximum speed-up is represented by the dashed line (y = x).

When using all available cores/CPUs of multiple-core processors, the execution time was up to 7 times quicker than the sequential task on the 8-cores Intel Xeon architecture, and 4 times quicker on the 4-cores Intel i7 architecture (Figure 1). With equal number of CPUs, the gain in computation time (speed-up) was lower on the 4 cores i7 processor (Figure 1).

In an ideal case, the speed-up brought by parallelization is linear with the number cores/CPUs allocated for the parallel task. Here, while the sequential inclusion of more cores/CPUs speeded up analysis time, speed-up did not increase linearly with the number of cores/CPUs allocated (Figure 1). The curve of speed-up vs. number of cores/CPUs reveals an inflexion point when all the available physical CPUs are used, i.e., 8 or 4 cores/CPUs on the Intel Xeon architecture or i7 architecture respectively. Even though in theory, the performance would increase linearly with the number of allocated cores/CPUs, this is not the case here. A possible reason for this is that the execution of a given individual job was slower when running along with other jobs than when running alone on the machine. This was noticeable on our example computer architecture, especially when using the hyperthreading. In the most extreme case, an individual STRUCTURE job took about 35% longer to execute when running along with 14 other jobs in parallel cores/CPUs, than when running alone. This was observed not only within the framework of *ParallelStructure*, but also when running manually several STRUCTURE jobs in command line. We thus conclude that this phenomenon is not caused by the implementation of *MPI_structure*() but it is rather inherent to the way the processor deals with distribution of execution resources, especially when hyperthreading.

The reduction in performance gain for each processor that is sequentially included in the analyses is not caused by excessive use of the available memory (RAM), as when the maximum numbers of cores/CPUs were allocated to the STRUCTURE jobs, the available RAM was never fully used on either computer. This is consistent with the fact that STRUCTURE is not known to be a memory-demanding program and thus extra performance gains would not be achieved through the addition of extra memory beyond the test specifications detailed here.

In addition to parallelization of structure runs, ParallelStructure offers the possibility to analyze large datasets, and divide analyses among combinations of populations without the need to manually produce multiple projects, as it is currently the case in STRUCTURE. However, because it relies on the command line version of STRUCTURE, ParallelStructure does not offer the possibility to set up repeated iterations for various values of parameter K, as it would be possible to do with the front-end version of STRUCTURE. In ParallelStructure, each iteration for each value of K has to be specified as a separated job on the ‘joblist’ input file. This issue was addressed by another freely available program: StrAuto (www.crypticlineage.net/pages/software.html) that streamlines command line analyses in STRUCTURE. This program makes it possible to set up multiple iterations for various values of parameter K from STRUCTURE command line version, however StrAuto is not parallelized yet, and does not seem to work on Windows operating system.

In conclusion, *ParallelStructure* provides population geneticists and molecular ecologists with an effective tool that is able to command STRCUTURE to run jobs in parallel in order to speed up computation time several-fold. It also provides a set of commands that enables large data sets to be analyzed in multiple ways without having to divide up populations into smaller data files. Based on the analysis of the example data, we conclude that the use of *MPI_structure*() or *parallel_structure*() can speed up computation time of genetic analysis in STRUCTURE, however, hyperthreading brings little improvement, and significant performance gain should not be expected when calling more processors than physically available on the machine.

### Software Availability

Software is hosted on R-forge (https://r-forge.r-project.org), and can be downloaded from: https://r-forge.r-project.org/R/?group_id=1636 or installed in R directly by typing: install.packages("ParallelStructure", repos="http://R-Forge.R-project.org")

Information document with installation instruction and example run can be downloaded from: http://parallstructure.r-forge.r-project.org/ParallelStructure.pdf

## Supporting Information

File S1 **User manual and installation instruction for R package ParallelStructure.**
(PDF)
