# Supplementary material for: ParallelStructure: A R Package to Distribute Parallel Runs of the Population Genetics Program STRUCTURE on Multi-Core Computers
Source: PLoS One. 2013 Jul 29;8(7):e70651. doi: 10.1371/journal.pone.0070651 (PMC3726640; doi:10.1371/journal.pone.0070651)
Supplement: File S1 — User manual and installation instruction for R package ParallelStructure. (PDF) [file pone.0070651.s001.pdf]

ParallelStructure

A package to provide R framework to run genetic analysis software STRUCTURE and make use of multi-core computers

1 INTRODUCTION

This Package provides a R framework to make use of multi-core computers when running analysis in the population genetics software STRUCTURE(*Pritchard, Stephens & Donnelly (2000)*). STRUCTURE is one of the most widely used population genetic software of the last decade. Introduced in year 2000, it has brought outstanding contribution to the field of population genetics by providing a user friendly tool for analyzing multi-locus genotype data to investigate population structure, hybridization, population admixture ...etc. Distributed as free software, `http://pritch.bsd.uchicago.edu/structure.html` the program comes in two versions: A user friendly graphic interface and a command line version. STRUCTURE analyses rely on multiple MCMC re-sampling and are thus often time consuming.
In general, one efficient way to speed up computing processes is to distribute tasks on several computing units (core/CPU). This solution imposes itself as the most common one since shared memory multi-core processors are now readily available on the market. Even common laptops are usually equiped with at least dual-core processors, and 4 to 8 core are becoming the norm. However, STRUCTURE does not support native multi-processor tasking. Nevertheless, it is possible to use STRUCTURE on multiple cores by simply opening several graphic-interface windows at the same time, or by using scripts to run STRUCTURE on the command line version and perform several STRUCTURE analysis simultaneously.
Opening several graphic windows remains a suboptimum solution as the user needs to to run manually each analysis after the previous one is done. It is thus a poorly automatized solution.
Using script programming can make a more efficient use of the multi-core processors by automatically distributing analyses to all available cores/CPUs and renewing the task of each core/CPU when a given job is completed. This solution is more efficient but requires specific script programming and parallel programming skills.

The present package provides a R framework to run genetic analysis in STRUCTURE and make efficient use of multi-core computers. It consists is a R script that imports STRUCTURE command line options into an R function, and run several STRUCTURE analysis in parallel by using either **Rmpi**

package (*MPI_structure* function) or **parallel** package (*parallel_structure* function).

2 CONTENT

The package consists in two main functions, *MPI_structure()* and *parallel_structure()* as well as an example data file and corresponding joblist file. The two functions *MPI_structure()* and *parallel_structure()* are equivalent as they perform the same task and work with the same input file and parameter set. The difference between them is the method that the function relies on for distributing jobs among CPUs: *MPI_structure()* relies on the R package **Rmpi** whereas *parallel_structure()* relies on R package **parallel**. **Rmpi** is distributed in the CRAN repository for MacOS, but will require manual installation under Windows (See section **install Rmpi for windows** in this document or from the **Rmpi** webpage `http://www.stats.uwo.ca/faculty/yu/Rmpi/`.. **Parallel** package is distributed with R since version 2.14.0 but is still under development. *parallel_structure()* might not be fully functional under Windows architecture, and should not be used in GUI or embedded environments as it may cause crashes. We thus strongly recommend to use *MPI_structure()* function by default in Windows. For Unix users, both *MPI_structure()* and *parallel_structure()* are available *parallel_structure()* would require R to run from the terminal.

A list of tasks to be performed is stored in a joblist file. In joblist file, each line corresponds to an individual job. While STRUCTURE input format requires a different dataset for each set of population, *ParallelStructure* offers the possibility for the user to work from a large input file containing all the populations one might need to analyze in STRUCTURE. Then, a set of "jobs" is defined in which all or only a subset of population can be included. This avoids making a different input file for each population subset. For each job, the user defines the set of populations to be included, STRUCTURE parameter K, burin and number of iteration. If all population in the data must be analyzed pairwise (*all VS all*), the list of populations for the given job can be replaced by "pairwaise.matrix" (see job T11 in example joblist)

3 USE

Standard documentation is available from R command

```
?ParallelStructure

?MPI_structure

?parallel_structure
```

MPI_structure and *parallel_structure* functions take many arguments, most of which are directly imported from STRUCTURE command line instructions. (see `http://pritch.bsd.uchicago.edu/software/readme_2_1/node33.html`

Ohter arguments are specific to **ParallelStructure** functions:

-*infile*: name of the datafile, eg: "structure_data"

-*outpath*: folder to write result files e.g "example/results/"

-*joblist*: name of the joblist file e.g "example/joblist1"

-*n_cpu*: number of core/CPU to be used for computation.

-*structure_path*: R must find the location of executable STRUCTURE command line file. this can be achieved in different ways:
Either in R: copy the location of STRUCTURE executable in a local variable *my_path* , eg: for MacOS
my_path="/Applications/Structure.app/Contents/Resources/Java/bin/"
or Windows *my_path="c:/Program Files (x86)/Structure2.3.4/bin/"*
Then, run the function in R with following arguments:

```
MPI_structure(structure_path=my_path,joblist='joblist1.txt',n_cpu=4,

  infile='example_data.txt',outpath='structure_results/',numinds=987,

numloci=9,printqhat=1)
```

Alternatively it is possible to make STRUCTURE executable file available from any place in the computer (in such case, set *structure_path*=NULL when calling the R function): On MacOS: copy the STRUCTURE executable command line from */Applications/Structure.app/Contents/Resources/Java/bin/* into *usr/local/bin* or on Windows, copy the address *c:/Program Files (x86)/Structure2.3.4/bin/* into the environment variable :
Right click "My Computer" and choose "Properties". Click "Advanced system

settings" to get a new window.
Click "Environment Variables. Locate variable "PATH"
Do not erase environment variables already present in the list. Add at the end of Variable value: ";c:/Program Files (x86)/Structure2.3.4/bin/"

3.1 Example

-1 call ParallelStructure in R:

```
library(ParallelStructure)
```

-2 make a directory to store result files called "structure_results"
in R for unix:

```
system('mkdir structure_results')
```

OR in R for Windows:

```
shell('mkdir structure_results')
```

-3 call example data file and joblisfile from the R package

```
data(structure_data)

data(structure_jobs)
```

-4 Write datafile and joblist file as text files in the working directory

```
write(t(structure_jobs),ncol=length(structure_jobs[1,]),file='joblist1.txt')

write(t(structure_data),ncol=length(structure_data[1,]),file='example_data.txt')
```

-5 Run STRUCTURE job with MPI_structure function:
If STRUCTURE executable is accessible from environment variables: Run

MPI_structure

```
MPI_structure(structure_path=NULL,joblist='joblist1.txt',n_cpu=4,

 infile='example_data.txt',outpath='structure_results/',numinds=987,

numloci=9,printqhat=1)
```

If STRUCTURE executable is NOT accessible from environment variables:
first define the location of STRUCTURE executable:

```
my_path="/Applications/Structure.app/Contents/Resources/Java/bin/"
```

OR

```
my_path="c:/Program Files (x86)/Structure2.3.4/bin/"
```

then run MPI_structure

```
MPI_structure(structure_path=my_path,joblist='joblist1.txt',n_cpu=4,
 infile='example_data.txt',outpath='structure_results/',numinds=987,
numloci=9,printqhat=1)
```

4 OUTPUTS

ParallelStructure will run STRUCTURE for all specified jobs in the joblist file, and write the output _f files in the directory specified in parameter "outpath". If parameters printqhat=1 or plot_output=1, _q files and graphs in .pdf format are also produced in the same directory. ParallelStructure also produces one .csv file called "results_summary" in the working directory. This file contains a table that summarises for each job listed in the joblist file: main job parameters (job ID, k, number of iteration and burnin, as well as result summary statistics log likelihood of the data, mean and variance of the log likelihood, and mean value of alpha)

5 INSTALL

5.1 MacOS

5.1.1 Rmpi

Install Rmpi from the CRAN repository, or follow the instruction in http://www.stats.uwo.ca/faculty/yu/Rmpi/ for manual installation.

5.1.2 ParallelStructure

Download binary file from http://r-forge.r-project.org/R/?group_id=1636 Then install ParallelStructure package from local .tar.gz archive by typing in R

```
install.packages("ParallelStructure_1.0.tar.gz",type='source')
```

OR from R-forge repository by typing in R:

```
install.packages("ParallelStructure", repos="http://R-Forge.R-project.org")
```

5.2 Windows

5.2.1 Rmpi

Rmpi for Windows is not available from CRAN repository. To install Rmpi, follow the next steps or look at the instruction from: `http://www.stats.uwo.ca/faculty/yu/Rmpi/` for manual installation.

1- Install MPICH2 for windows (32 or 64 bit depending on the R version you are using) `http://www.mpich.org/downloads/`

During installation you will have to specify a pass phrase, keep the default "behappy" or change it for a new one.

2- Go to your program files (or program files(x86) if you installed 32 bit version). search for MPICH2 folder and run *wmpiregister*. You need to register your username *id* and *password* for your computer session. click on "register" and "OK"

3- Add MPICH2 bin directory to PATH environment variable:
Right click "My Computer" and choose "Properties". Click "Advanced system settings" to get a new window.
Click "Environment Variables. Locate variable "PATH"
Do not erase environment variables already present in the list. Add at the end of Variable value:

```
";C:\Program Files\MPICH2\bin"
```

```
(OR ";C:\Program Files x(86)\MPICH2\bin" if you installed 32 bit version)
```

4- open "command prompt" as administrator. make sure you are on "C" drive. (if not, type "c:" to change drive), then type
smpd -install -phrase behappy (or change "behappy" by the passphrase you entered in step 1)

then type

smpd -status

it should return "smpd running on "hostname" "

5- Download Rmpi for Windows here: `http://www.stats.uwo.ca/faculty/yu/Rmpi/`

Launch R with administrator to install Rmpi. Use the option "install from local *.zip* file"

Quit R and launch R with normal user. Try the following codes to see if Rmpi runs properly

```
library(Rmpi)
mpi.spawn.Rslaves()
mpi.parReplicate(20, mean(rnorm(1000000)))
```

```
mpi.close.Rslaves()
```

5.2.2 ParallelStructure

Download binary file from `http://r-forge.r-project.org/R/?group_id=1636`
Then install ParallelStructure package from local .zip archive by typing in R

```
install.packages("ParallelStructure_1.0.zip",repos=NULL)
```

OR from R-forge repository by typing in R:

```
install.packages("ParallelStructure", repos="http://R-Forge.R-project.org")
```

5.3 Linux

5.3.1 Rmpi

Rmpi for Linux is not available from CRAN repository. To install Rmpi, follow the instructions from: `http://www.stats.uwo.ca/faculty/yu/Rmpi/` for manual installation. **However, because Linux runs R from Terminal**

by default, Linux users can simply install ParallelStructure package without Rmpi, and use the function *parallel_structure()* instead of *MPI_structurte()* . This only requires R version 2.14 or later.

5.3.2 ParallelStructure

Download binary file from `http://r-forge.r-project.org/R/?group_id=1636`
Then install ParallelStructure package from local .tar.gz archive by typing in R

```
install.packages("ParallelStructure_1.0.tar.gz",type='source')
```

OR from R-forge repository by typing in R:

```
install.packages("ParallelStructure", repos="http://R-Forge.R-project.org")
```

6 WARNING: USE OF POPINFO

Using ParallelStructure functions with usepopinfo=1 may produce STRUCTURE output files with a column of population IDs that does not match the initial input data file. Details and possible solutions

are given bellow:

In STRUCTURE, a column of population IDs (pop_ID) can be included in the input file (see option POPDATA). If USEPOPINFO=0, pop_ID is not used as prior information to assist clustering, pop_ID is then just a convenient information to separate the individuals in the output graph, and can contain any user defined list of integers as population identificator. However, if USEPOPINFO=1, pop_ID is used as prior information to assist clustering, **STRUCTURE will need that the integers in pop_ID are equal or smaller than K. e.g., if STRUCTURE runs with parameter K=3, pop_ID can only contain integers from 1 to 3.**
ParallelStructure offers the possibility to analyse large datasets in multiple ways without need for the user to split the data manually and generate multiple sub-datasets. The scripts in ParallelStructure will generate these sub-datasets automatically.
For example: if the main dataset contains five populations, and pop_ID is a column of integers from 1 to 5,
pop_ID
1
1
...
2
2
...
5

and the analyses to be performed are stated in the joblist file as:
T1 1,2,3 3 1000 10000
T2 3,4,5 3 1000 10000

Job T1 will consist in the analysis of populations 1,2 and 3
Job T2 will consist in the analysis of populations 3,4 and 5

ParallelStructure will automatically generate one sub-datafile for each job: with population 1,2 and 3 for job T1, and with populations 3,4 and 5 for job T2. For job T2 however, pop_ID will need to be converted and IDs 3,4 and 5 will be converted into 1,2 and 3 in order to match STRUCTURE's requirements that pop_ID contains only integers from 1 to n where n is equal or smaller than K. **When USEPOPINFO=1, this conversion is done automatically by ParallelStructure scritps.** The output files (_f and _q) contain the converted pop_IDs wich are different from the original data file pop_IDs. The user can chose bertween two options to deal with pop_IDs:

1- Using ParallelStructure functions with option **revert_convert=1**. With this option, ParallelStructure will import STRUCTURE output files (_q and _f) in R, change the population IDs into the original ones and write the files back

into the "outpath" directory. During that process, STRUCTURE output files are slightly modified (number of space separators in the individual assignment table). We would thus warn the users that revert_convert=1 might be a source of bugg when using resource programs such as CLUMPP or Structure harvester to analyse STRUCTURE outputs. The function was tested on the example data file with USEPOPINFO=1 and REVERT_CONVERT=1. The resulting _f and _q files were successfully uploaded into structure harverster, however, other data files might not work as well. We invite the users to be careful and report such bugg to the authors

2- Doing nothing: (using ParallelStructure functions with default option **revert_convert=0**) This is the best way to preserve STRUCTURE output files untouched and ready to be imported into resource programs such as CLUMPP or Structure harvester. Using a column of individuals IDs that also contain a population indicator (e.g. pop1.1, pop1.2 ... pop2.1, pop2.2 etc...) is also a convenient way to keep easily interpretable output files.
